# Nutrient management via organic and organomineral fertilization modulates yield and rosmarinic acid accumulation in *Melissa officinalis* L.

**DOI:** 10.3389/fpls.2026.1845091

**Published:** 2026-05-08

**Authors:** Merve Göre, Ayşe Betül Avcı, Onur Bayız, Bihter Çolak Esetlili, Bintuğ Öztürk

**Affiliations:** 1 Department of Medicinal and Aromatic Plants, Ödemiş Vocational School, Ege University, Izmir, Türkiye; 2 Department of Soil Science and Plant Nutrition, Faculty of Agriculture, Ege University, Izmir, Türkiye; 3 Department of Pharmaceutical Botany, Faculty of Pharmacy, Ege University, Izmir, Türkiye

**Keywords:** essential oil, herbage yield, lemon balm, nutrient management, plant nutrition, secondary metabolism

## Abstract

**Introduction:**

Optimizing fertilization strategies in *Melissa officinalis* L. requires an integrated assessment of biomass production and secondary metabolite synthesis.

**Methods:**

This study evaluated the effects of organic and organomineral fertilization on herbage yield, essential oil (EO) yield, and leaf rosmarinic acid (RA) content under field conditions in Ödemiş (Türkiye) during the warm and dry 2024 growing season. A split-plot design was used, with two cultivars (Melis and Quedlinburger) assigned as main plots and six fertilization treatments (G0–G5) as subplots, evaluated across two harvest times (H1 and H2).

**Results:**

Fertilization significantly affected all parameters (p < 0.01). The highest fresh herbage yield was obtained with G3 (ammonium sulfate combined with humic and fulvic acids) at H1, reaching 15.41 t ha^-^¹ in Quedlinburger. Organomineral fertilization (G5; 12-12-12, 50% organic matter) maximized EO yield at H1, reaching 118.10 L ha^-^¹ in Melis, and maintained higher mean RA content than the control across both harvests (62.47 and 53.41 mg g^-^¹ at H1 and H2, respectively; mean across cultivars), corresponding to 69–99% higher RA content than the control. A marked phenological shift was observed, with EO content declining by ~50% from H1 to H2, while RA content also decreased by ~20% across harvests but remained highest under G5 treatment, which may be associated with changes in carbon allocation between the terpenoid (MEP) and phenylpropanoid pathways. Multivariate analysis supported these findings, with principal component analysis explaining 88.2% of total variance and clustering identifying G5 and G3 as the most effective treatments.

**Discussion:**

Overall, integrated nutrient management enhanced both yield and phytochemical quality under heat and water-deficit conditions.

## 1 Introduction

The global market for medicinal and aromatic plants (MAPs) has expanded markedly over the past two decades, driven by increasing demand for plant-based therapeutics, functional foods, and natural cosmetic products (Zamani et al., 2025). Within this context, *Melissa officinalis* L. (lemon balm; Lamiaceae), a species native to the Mediterranean basin and widely naturalized across temperate regions, has gained considerable economic and pharmacological importance. Its value derives from a dual secondary metabolite system comprising volatile terpenoids, primarily geranial (citral-a) and neral (citral-b), collectively defined as “citral” and phenolic compounds, most notably RA. Accordingly, the European Pharmacopoeia Commission (2023) defines quality standards for *Melissa folium* based on both EO composition (≥ 40% citral) and RA content (≥ 1.0% dry weight), highlighting the need for integrated production strategies that ensure both yield and phytochemical quality.

In Türkiye, production of *M. officinalis* fresh herb has increased rapidly, rising from 93 t in 2020 to 266 t in 2022 (Turkish Statistical Institute, 2023), reflecting its growing role in the herbal tea, extract, and EO industries (Bağdat and Coşge, 2006; Carocho et al., 2015; Krivokapić, 2025). Despite this expansion, agronomic optimization remains limited, particularly with respect to the simultaneous management of biomass yield, EO productivity, and RA accumulation under Mediterranean field conditions. Existing studies have largely focused on single quality parameters or conventional inorganic fertilization approaches (Wiśniowska-Kielian and Lorenc-Kozik, 2016; Mafakheri et al., 2016) while the role of integrated nutrient management strategies, including organomineral fertilizers and humic substance-based inputs has received comparatively little attention. Previous research indicates that fertilization can significantly influence plant nutrient status, biomass production, and EO composition (Yetgin, 2010; Tepecik et al., 2016; Fallah et al., 2024a) and similar responses have been reported in other Lamiaceae species where fertilizer type and harvest timing strongly affect EO yield. In *Salvia officinalis*, essential oil yield and composition were significantly influenced by harvest stage, harvest time, and nitrogen applications (Soltanbeigi and Samadpourrigani, 2021; Hazrati et al., 2022). Comparable fertilizer-mediated responses have also been reported in *Mentha piperita*, where nitrogen application and vermicompost-based integrated treatments enhanced both essential oil content and yield (Bidgoli and Mahdavi, 2018; Ostadi et al., 2020; Asadi et al., 2023). In *Thymus vulgaris*, organic fertilization significantly increased thymol content and productivity, while mineral and organomineral treatments resulted in higher yields than organic or control treatments (Raffo et al., 2023; Honorato et al., 2024). Moreover, organic fertilization has been associated with improvements in growth and phytochemical profiles in medicinal plants (Rostaei et al., 2024). However, a comprehensive evaluation linking nutrient management strategies to both yield formation and metabolic regulation in *M. officinalis* remains lacking.

Terpenoids are synthesized via two distinct pathways: the cytosolic mevalonate (MVA) pathway and the plastidial methylerythritol phosphate (MEP) pathway. Together with the phenylpropanoid pathway, these metabolic routes are interconnected through carbon flux originating from primary metabolism, and their interaction under different nutrient regimes represents a key mechanism governing yield-quality relationships in MAPs (Gershenzon, 1994; Degenhardt et al., 2009). Nutrient availability can regulate carbon partitioning between these pathways, thereby influencing both EO biosynthesis and phenolic compound accumulation (Herms and Mattson, 1992; Nguyen and Niemeyer, 2008; Wang et al., 2023). Organic and organomineral fertilizers, characterized by gradual nutrient release and their positive effects on soil water retention, microbial activity, and micronutrient availability, may play a critical role in modulating these processes, particularly under the warm and drought-prone conditions typical of Mediterranean environments (Nardi et al., 2002; Tejada et al., 2006; Canellas et al., 2015). Similar responses have been observed across various Lamiaceae species, where organic fertilization significantly enhances both biomass production and essential oil yield. Organic fertilization with vermicompost in *Mentha spicata* not only increased yield but also enriched the oil with total polyphenols and carvone, thereby improving its antioxidant and medicinal properties (Meloni et al., 2019). In *Melissa officinalis*, the application of organic manures has been shown to increase biomass by 41–60% and essential oil yield by 60–71% (Fallah et al., 2024b). Studies on *Origanum onites* revealed that organic fertilization, particularly with farmyard manure and spent mushroom compost, increased fresh yield by up to 13.59% and boosted essential oil yield by 18.8–50.1% compared to untreated controls (Asri, 2023). Furthermore, in *Salvia fruticosa*, organic regimes have achieved significant essential oil yields ranging from 291.54 to 407.10 L ha^-^¹ (Asri et al., 2023). These findings support the role of organic inputs as sustainable agronomic tools that improve soil physical and chemical properties while promoting the accumulation of high-quality secondary metabolites (Lopes et al., 2019).

RA is the principal phenolic compound in *M. officinalis* and a key pharmacological marker due to its antioxidant, antiviral, anti-inflammatory, and anxiolytic properties (World Health Organization (WHO), 2002; Shakeri et al., 2016). As a product of the phenylpropanoid pathway, its biosynthesis is regulated by phenylalanine ammonia-lyase (PAL) and depends on carbon flux through phenylalanine-derived metabolism (Petersen et al., 2010). According to the carbon and nutrient balance (CNB) hypothesis, phenolic compounds such as RA tend to accumulate under conditions of balanced but non-excessive nitrogen availability, where surplus carbon is preferentially allocated to secondary metabolism rather than primary growth (Gershenzon, 1994; Bryant et al., 1983). Accordingly, RA concentration is highly sensitive to fertilization regime, harvest timing, and water availability (Habibi Sharafabad et al., 2022; Beyk-Khormizi et al., 2023). However, the extent to which RA accumulation and EO production are co-regulated under organic and organomineral fertilization remains insufficiently understood.

This study aimed to evaluate the effects of six fertilization regimes on herbage yield, EO content and yield, and RA accumulation in two *M. officinalis* cultivars across two harvest times under the anomalously warm and dry conditions of the 2024 growing season, which was recorded as the warmest year in the last 54 years in Türkiye, with below-average precipitation in the Aegean Region (Turkish State Meteorological Service, 2025). Multivariate approaches, including principal component analysis (PCA) and hierarchical cluster analysis (HCA), were employed to integrate agronomic and phytochemical responses. The study specifically aimed to determine whether nutrient-driven metabolic regulation enables the co-optimization of EO yield and RA accumulation or leads to a trade off between these key quality parameters.

## 2 Materials and methods

### 2.1 Experimental site and plant material

**Figure 1:**
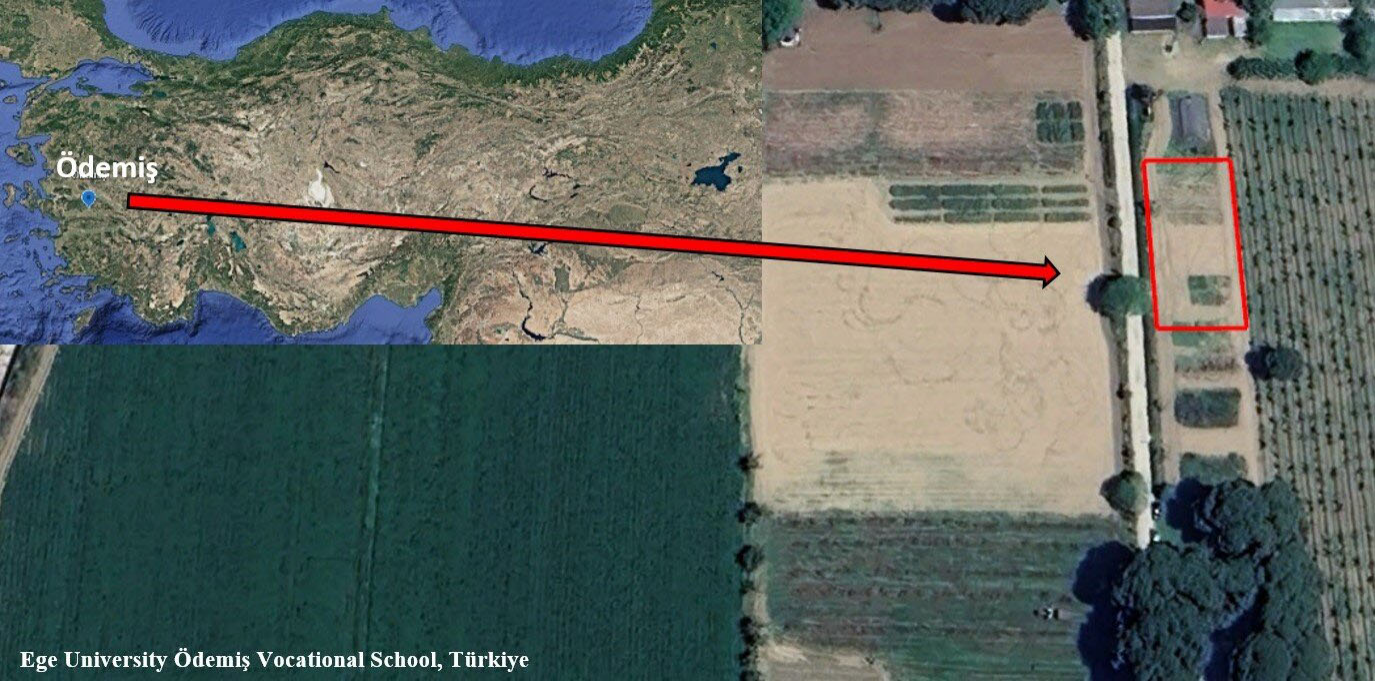
Experimental site.

The experiment was conducted at the research field of Ege University Ödemiş Vocational School (38°13′10″N, 27°57′50″E; 90 m above sea level) in Ödemiş, İzmir Province, Türkiye, characterized by a typical Mediterranean climate with hot, dry summers (**Figure 1**). Two *Melissa officinalis* L. cultivars were used: “Melis” (M1), a registered Turkish cultivar obtained from the Menemen Agricultural Research Institute, and “Quedlinburger” (M2), a certified German cultivar (Quedlinburger Saatgut, Germany). Seedlings were initially raised in a nursery and transplanted to the experimental field in April 2022 at a spacing of 70 × 30 cm. The 2024 growing season corresponded to the third productive year of the perennial stand, ensuring stabilized plant establishment and consistent yield performance. Fertilization treatments were initiated in the second productive year and continued through the third productive year (2024). The first productive year served as an establishment phase, with no fertilization inputs applied, ensuring uniform baseline conditions across all plots.

### 2.2 Climatic conditions of the experimental site

**Figure 2:**
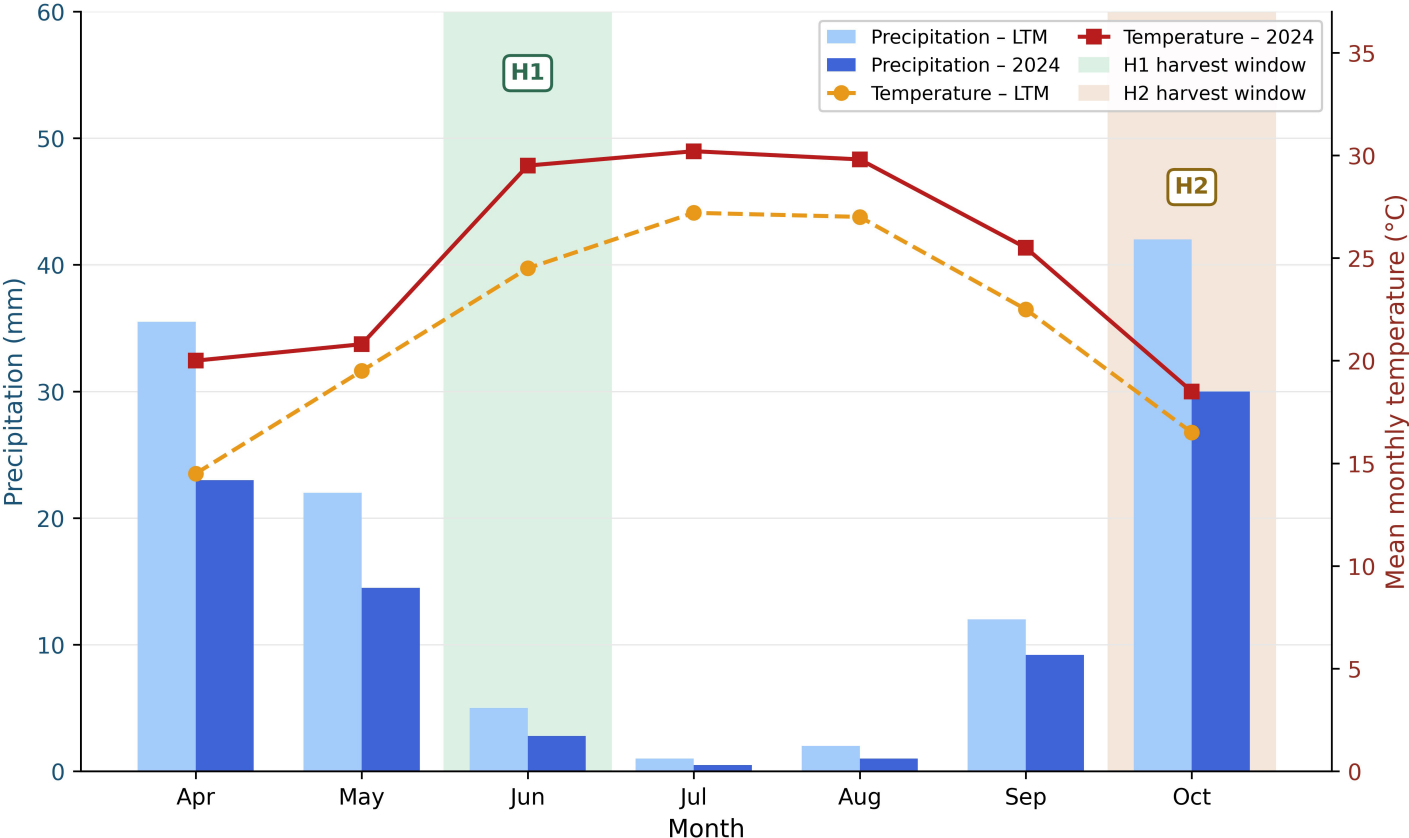
Monthly mean temperature (°C) and total precipitation (mm) at Ödemiş, İzmir during the *M. officinalis* growing season (April–October 2024 vs. 1991–2020 long-term mean, LTM). Green and orange shaded areas indicate the approximate harvest windows: H1, first harvest; H2, second harvest.

The Ödemiş experimental site is characterized by a typical Mediterranean climate, with hot, dry summers and mild, wet winters (**Figure 2**). Mean monthly temperatures increase from winter minima in January-February to peak summer values exceeding 27°C in July-August, while precipitation is largely confined to autumn and winter, with negligible rainfall during the May-August period. The 2024 growing season deviated markedly from these long-term patterns. According to the Turkish State Meteorological Service, (2025), 2024 was the warmest year on record in Türkiye, with a mean annual temperature anomaly of +1.7°C relative to the 1991–2020 long-term mean (LTM). In the Aegean Region, key months during the *M. officinalis* growth period exhibited substantial positive anomalies, including April (+4.1 °C), June (+3.5 C), and July (+2.5°C), while May was slightly cooler than the LTM (-0.6°C). Annual precipitation was 22.4% below the LTM, with pronounced deficits in April (-34%), June (-40%), and July (-58%), coinciding with the main vegetative growth phase and the H1 pre-flowering harvest period.

These concurrent heat and water-deficit conditions likely imposed significant constraints on soil moisture availability and plant water status, thereby influencing nutrient uptake, carbon assimilation, and secondary metabolite biosynthesis during the experimental period. Such stress conditions are particularly relevant for interpreting fertilization responses, as nutrient availability and metabolic allocation are strongly regulated by plant water and thermal status.

### 2.3 Soil characterization

Soil samples were collected from the experimental plots prior to treatment establishment at two depths (0–30 cm and 30–60 cm) and analyzed for selected physical, chemical, and nutritional properties (**Table 1**). The soil was characterized by a high sand content (77–79%), classified as sandy loam in the surface horizon and loamy sand in the subsoil according to USDA criteria. This coarse texture indicates low water-holding capacity and high hydraulic conductivity, conditions that increase susceptibility to water deficit and nutrient leaching under Mediterranean environments.

**Table 1:** Physical, chemical, and nutritional properties of the experimental soil.

Soil property	0–30 cm	Classification	30–60 cm	Classification
Sand (%)	77.52		79.52	
Silt (%)	13.64		13.64	
Clay (%)	8.84		6.84	
Texture class	Sandy loam		Loamy sand	
CaCO_3_ (%)	0.56	Low lime	0.80	Low lime
pH	7.14	Neutral	7.39	Slightly alkaline
EC (dS m^-^¹)	0.188	Non-saline	0.106	Non-saline
Organic matter (%)	1.036	Low	0.796	Very low
Total N (%)	0.056	Medium	0.056	Medium
Available P (mg kg^-^¹)	8.6	Medium	8.2	Medium
Available K (mg kg^-^¹)	75.4	Deficient	65.98	Deficient
Available Ca (mg kg^-^¹)	950	Low	570	Very low
Available Mg (mg kg^-^¹)	556	Sufficient	482	Sufficient
Available Fe (mg kg^-^¹)	7.45	Adequate	5.15	Adequate
Available Mn (mg kg^-^¹)	5.52	Sufficient	3.32	Sufficient
Available Zn (mg kg^-^¹)	1.01	Adequate	0.53	Borderline deficient
Available Cu (mg kg^-^¹)	1.10	Sufficient	0.65	Sufficient

Soil reaction was neutral to slightly alkaline (pH 7.14–7.39), while organic matter content was low (0.80–1.04%) in both layers, indicating limited intrinsic nutrient buffering capacity. Available potassium concentrations ranged from 66 to 75 mg kg^-^¹, and available calcium from 570 to 950 mg kg^-^¹. Micronutrient levels were generally adequate for Fe, Mn, and Cu, whereas Zn concentrations were marginal at the 30–60 cm depth.

Soil analyses were performed using standard methods for pH, organic matter, and nutrient determination (Page, 1982).

### 2.4 Experimental design and fertilization treatments

All fertilization treatments were calculated on an equivalent basis of 60 kg N ha^-^¹, taking into account the nitrogen requirement of *M. officinalis* (Baydar, 2016) and the initial soil total N contents. Control plots (G0) received no fertilization. The organomineral fertilizer treatment (G5) was applied as a single dose. In mineral-based treatments (G1 and G3), a basal application of compound fertilizer (15-15-15) was applied as a basal dressing to supply one-third of the total N requirement, and two-thirds N was supplemented with ammonium sulfate applied as a single dose. In treatments including organic amendments (G2 and G4), well-decomposed cattle manure was incorporated into the soil as a basal application. The manure was chemically characterized prior to application and contained 0.41% total N, 0.10% P_2_O_5_, 0.50% K_2_O, 0.20% Na, 0.90% Ca, and 0.45% Mg, together with 400 mg kg^-^¹ Fe, 320 mg kg^-^¹ Mn, 55 mg kg^-^¹ Cu, and 62 mg kg^-^¹ Zn. The remaining N requirement was then supplemented with ammonium sulfate. For treatments receiving humic substances (G3 and G4), humic and fulvic acids (containing 20% total humic + fulvic acid) were applied single dose at a rate of 5 L ha^-^¹ via drip irrigation water approximately 10 days after N fertilization (**Table 2**). This approach ensured that all treatments, except the control, received equivalent N inputs while allowing the evaluation of different nutrient sources and management strategies.

**Table 2:** Fertilizer treatments applied in the experiment.

Code	Treatment description	Total N applied (kg N ha-1)
G0	Control (unfertilized)	–
G1	15-15-15 + Ammonium sulfate (AS)	32 (10.7 + 21.3)
G2	Animal manure (10 t ha^-^¹) + AS	32 (6.2 + 25.8)
G3	15-15-15 + AS + Humic & fulvic acids	32 (10.7 + 21.3)
G4	Animal manure + AS + Humic & fulvic acids	32 (6.2 + 25.8)
G5	Organomineral fertilizer (12-12-12, 50% organic matter)	32

### 2.5 Harvest procedure and measured parameters

Two harvests were conducted in 2024: H1 (June) and H2 (September). Shoots were cut at approximately 10 cm above the soil surface. Plant height was measured from the soil surface to the apex using 10 randomly selected plants per subplot immediately before each harvest. Fresh herbage yield was determined by harvesting all inner-row plants within each subplot, weighing them, and converting the data to a per hectare basis. Dry herbage yield was calculated from a 500 g representative subsample dried at 35°C for 72 h and expressed as kg ha^-^¹. Leaf ratio was determined on a fresh weight basis by manually separating leaves from stems of a 500-g fresh subsample immediately after harvest; fresh and dry leaf yields were calculated accordingly.

EO content (% DW) was determined by hydrodistillation of 200 g dried herbage using a Neo-Clevenger (2.5 L water, 2–3 h) following European Pharmacopoeia Commission (2023) procedures. EO yield (L ha^-^¹) was calculated by multiplying EO content by dry herbage yield.

RA content (mg g^-^¹ DW) was determined by HPLC-DAD (Agilent 1260 Infinity II, Waldbronn Germany) analysis of methanolic leaf extracts. Dried leaf material (0.5 g DW) was extracted with 10 mL of 80% (v/v) aqueous methanol by sonication for 30 min at 40°C, followed by centrifugation and filtration through a 0.45 μm membrane filter. Chromatographic separation was performed on a C18 column (250 × 4.6 mm, 5 μm) using a gradient mobile phase of 0.1% formic acid in water (A) and acetonitrile (B) at a flow rate of 1.0 mL min^-^¹; detection was at 330 nm. Quantification was performed using a six-point external calibration curve of rosmarinic acid standard (≥ 96% purity, Sigma-Aldrich; range: 5–200 μg mL^-^¹; R² > 0.999). The limit of detection (LOD) and limit of quantification (LOQ) were 1.6 and 4.9 μg mL^-^¹, respectively, consistent with previously validated HPLC-DAD methods for rosmarinic acid quantification in Melissa officinalis (Arceusz and Wesolowski, 2013; Caleja et al., 2017). All samples were analyzed in three replicates (n = 3).

### 2.6 Statistical analysis

Data from each harvest (H1 and H2) were analyzed separately to account for phenological differences between harvest stages. A split-plot analysis of variance (ANOVA) was performed, with cultivars assigned to main plots and fertilizer treatments to subplots. Mean comparisons were conducted using Duncan’s multiple range test at p < 0.05 and p < 0.01. Prior to analysis, data normality and homogeneity of variance were verified using the Shapiro–Wilk and Levene’s tests, respectively. All ANOVA analyses were performed using SPSS v. 26.0 (IBM Corp, 2019). Principal component analysis (PCA) was conducted on a complete dataset consisting of 24 observations (6 treatments × 2 cultivars × 2 harvests) and six variables (plant height, dry herbage yield, dry leaf yield, EO content, EO yield, and RA content) Prior to analysis, all variables were mean-centered and standardized to unit variance (z-scores) to eliminate scale effects arising from differences in measurement units. PCA was then performed based on the correlation matrix. Hierarchical cluster analysis (HCA) was applied to treatment mean values (n = 6) using Ward’s linkage method and squared Euclidean distance. PCA and HCA were performed using the scikit-learn library in Python v. 3.11 (Pedregosa et al., 2011).

## 3 Results

### 3.1 Plant height

At H1, plant height ranged from 37.20 cm (G1) to 45.90 cm (G4 and G5). Fertilization treatments significantly affected plant height, with G4 and G5 producing taller plants compared to G0 and G1 (**Table 3**). The cultivar effect was also significant, with “Melis” exhibiting slightly greater plant height than “Quedlinburger” (42.53 vs. 41.76 cm; F = 7.31, p < 0.05). The cultivar × treatment interaction was not significant (F = 1.11, p > 0.05), indicating similar responses of both cultivars to fertilization treatments at H1.

**Table 3:** Plant height (cm) of *M. officinalis* as affected by fertilization treatments at two harvest times (H1 and H2) for the cultivars ‘Melis’ and ‘Quedlinburger’.

Treatment	H1 Melis (cm)	H1 Quedl. (cm)	H1 Mean	H2 Melis (cm)	H2 Quedl. (cm)	H2 Mean
G0	40.53 ± 2.71	40.8 ± 2.71	40.67 ± 2.23 b	25.2 ± 2.86 h	37.67 ± 7.25 c	31.43 ± 4.12 d
G1	37.23 ± 6.34	37.17 ± 6.34	37.20 ± 4.14 c	32.53 ± 3.98 e	35.83 ± 2.69 d	34.18 ± 2.91 cd
G2	41.4 ± 3.29	41.4 ± 4.10	41.4 ± 3.19 b	30.87 ± 4.49 fg	31.2 ± 4.49 ef	31.03 ± 2.21 de
G3	43.43 ± 2.44	40.37 ± 2.06	41.90± 2.14 b	31.43 ± 3.12 ef	40.27 ± 4.67 b	35.85 ± 3.65 b
G4	45.9 ± 4.63	45.9 ± 4.63	45.9 ± 4.63 a	29.6 ± 1.19 g	30.93 ± 3.88 fg	30.27 ± 2.19 e
G5	46.67 ± 2.14	44.93 ± 0.96	45.80 ± 2.23a	31.87 ± 6.37 ef	41.87 ± 2.28 a	36.87 ± 2.09 a
Mean	42.53 ± 3.10 a	41.76 ± 4.15 b	42.14 ± 2.56	30.25 ± 1.98 b	36.29 ± 3.11 a	33.27 ± 2.02
F Cultivar	7.31*			13.07**		
F Treatment	27.00**			162.68**		
F Cult×Treat	1.11 ns			136.57**		

*Different letters within each harvest indicate significant differences (Duncan’s test, p < 0.05). ns, not significant; *p < 0.05; **p < 0.01. Quedl, Quedlinburger; n, 3.

At H2, plant height decreased compared to H1, reflecting regrowth after the first harvest. In contrast to H1, “Quedlinburger” exhibited significantly greater plant height than “Melis” (36.29 vs. 30.25 cm; F = 13.07, p < 0.01). Among treatments, G5 produced the highest regrowth (36.87 cm), whereas G4 resulted in the lowest values (30.27 cm). The cultivar × treatment interaction was highly significant (F = 136.57, p < 0.01), indicating differential responses of the two cultivars under the second harvest conditions.

### 3.2 Fresh herbage yield

At H1, fresh herbage yield ranged from 9.81 t ha^-^¹ (G0) to 13.91 t ha^-^¹ (G3 treatment mean), with “Quedlinburger” under G3 achieving the highest individual value (15.41 t ha^-^¹; **Table 4**). Cultivar effect was significant at H1, with “Quedlinburger” producing higher yields than “Melis” (12.60 vs. 11.82 t ha^-^¹; F = 197.86, p < 0.01). All fertilization treatments resulted in significantly higher yields compared to the control (G0).

**Table 4:** Fresh herbage yield (t ha^-^¹) of *M. officinalis* as affected by fertilization treatments at two harvest times (H1 and H2) for the cultivars ‘Melis’ and ‘Quedlinburger’.

Treatment	H1 Melis (t ha^-^¹)	H1 Quedl. (t ha^-^¹)	H1 Mean	H2 Melis (t ha^-^¹)	H2 Quedl. (t ha^-^¹)	H2 Mean
G0	9.51 ± 1.71 e	10.11 ± 1.14 e	9.81 ± 1.10 e	6.38 ± 0.98 ef	7.65 ± 0.99 bcd	7.02 ± 0.01 c
G1	12.23 ± 1.88 c	12.27 ± 1.33 c	12.25 ± 1.91c	5.79 ± 0.71f	9.20 ± 1.01a	7.50 ± 0.61ab
G2	12.62 ± 1.61 c	13.58 ± 2.02 b	13.10 ± 1.76 b	6.48 ± 0.78 e	2.37 ± 0.40 h	4.43 ± 0.33 e
G3	12.41 ± 1.22 c	15.41 ± 2.46 a	13.91 ± 2.21a	8.14 ± 1.01bc	6.48 ± 0.51e	7.31 ± 0.44 bc
G4	11.36 ± 1.11 d	11.36 ± 1.55 d	11.36 ± 2.02 d	7.54 ± 0.91cd	3.27 ± 0.27 g	5.41 ± 0.66 d
G5	12.82 ± 1.02 bc	12.85 ± 1.77 bc	12.83 ± 1.98 b	7.18 ± 0.80 d	8.18 ± 0.67 b	7.68 ± 0.79 a
Mean	11.82 ± 1.76 b	12.59 ± 1.19 a	12.20± 1.86	6.92 ± 0.90 a	6.19 ± 0.58 b	6.55± 0.71
F Cultivar	197.86**			76.58**		
F Treatment	193.63**			258.86**		
F Cult×Treat	30.76**			361.04**		

Different letters within each harvest indicate significant differences (Duncan's test, p < 0.05). ** indicates significance at p < 0.01. Quedl. = Quedlinburger, n = 3.

At H2, overall yield levels decreased compared to H1, reflecting regrowth conditions following the first harvest. In contrast to H1, “Melis” significantly outperformed “Quedlinburger” (6.92 vs. 6.19 t ha^-^¹; F = 76.58, p < 0.01). Among treatment × cultivar combinations, the lowest yield was observed in G2–Quedlinburger (2.37 t ha^-^¹), whereas the corresponding control (G0–Quedlinburger) produced 7.65 t ha^-^¹. The highest mean yield at H2 was obtained under G5 (7.68 t ha^-^¹).

The cultivar × treatment interaction was highly significant at both harvests (p < 0.01), indicating differential responses of the two cultivars to fertilization treatments.

### 3.3 Dry herbage and leaf yields

Dry herbage yield followed patterns similar to those observed for fresh herbage (**Table 5**). At H1, the highest values were obtained in G3 (2.80 t ha^-^¹) and G5 (2.69 t ha^-^¹), whereas G0 showed the lowest yield (1.83 t ha^-^¹).

**Table 5:** Dry herbage yield (t ha^-^¹) of *M. officinalis* as affected by fertilization treatments at two harvest times (H1 and H2) for the cultivars ‘Melis’ and ‘Quedlinburger’.

Treatment	H1 Melis (t ha^-^¹)	H1 Quedl. (t ha^-^¹)	H1 Mean	H2 Melis (t ha^-^¹)	H2 Quedl. (t ha^-^¹)	H2 Mean
G0	1.83 ± 0.56 d	1.83± 0.21 d	1.83± 0.44 d	1.34 ± 0.21 ef	1.41± 0.14 e	1.37± 0.05 c
G1	2.10 ± 0.21 cd	2.13± 0.13 cd	2.12± 0.23 c	1.27 ± 0.19 fg	1.85± 0.28 c	1.56± 0.10 b
G2	2.75 ± 0.67 a	2.75± 0.65 a	2.75± 0.41 ab	1.20± 0.14 g	0.56± 0.16 h	0.88± 0.11 e
G3	2.64± 0.71 ab	2.96± 0.68 a	2.80± 0.46 a	2.19± 0.30 a	1.44± 0.20 de	1.81± 0.18 a
G4	2.29± 0.54 bc	2.75± 0.22 a	2.52± 0.38 b	1.96± 0.28 bc	0.56± 0.08 h	1.26 ± 0.22 d
G5	2.58± 0.33 ab	2.79± 0.12 a	2.69± 0.29 ab	2.02± 0.18 b	1.56± 0.09 d	1.79 ± 0.19 a
Mean	2.36± 0.42 b	2.53± 0.30 a	2.45± 0.21	1.66± 0.11e	1.23± 0.10	1.45± 0.10
F Cultivar	8.50*			2800.31**		
F Treatment	49.00**			398.25**		
F Cult×Treat	2.98*			378.30**		

*Different letters within each harvest indicate significant differences (Duncan’s test, p < 0.05). ns, not significant; *p < 0.05; **p < 0.01. Quedl, Quedlinburger; n, 3.

At H2, overall yield levels decreased compared to H1. “Melis” produced significantly higher dry herbage yields than “Quedlinburger” (p < 0.05), with the highest value observed in G3 for “Melis” (2.19 t ha^-^¹). The lowest yield at H2 was recorded in G2–Quedlinburger (0.56 t ha^-^¹).

Leaf ratio remained relatively stable across treatments at both harvests, ranging between 74% and 77% (**Table 6**). Cultivar effect was significant, with “Melis” consistently exhibiting a higher leaf ratio than “Quedlinburger” (F = 147.00, p < 0.01 at H1; F = 3.27, p < 0.05 at H2). Treatment effect was significant at H1 but not at H2 (F = 0.14, p > 0.05).

**Table 6:** Leaf ratio (%) of *M. officinalis* as affected by fertilization treatments at two harvest times (H1 and H2) for the cultivars ‘Melis’ and ‘Quedlinburger’.

Treatment	H1 Melis (%)	H1 Quedl. (%)	H1 Mean	H2 Melis (%)	H2 Quedl. (%)	H2 Mean
G0	76.67± 2.10	76.00± 3.09	76.33± 2.87	77.33± 4.16	74.00± 2.67	75.67± 1.15
G1	77.00± 3.13	75.33± 2.86	76.17± 3.04	76.00± 2.32	76.00± 2.19	76.00± 1.61
G2	76.00± 1.22	74.67± 2.47	75.33 ± 2.32	75.33± 3.90	75.33± 3.01	75.33± 3.09
G3	76.00± 2.16	75.33± 3.02	75.67± 2.66	76.00± 3.65	74.67± 2.89	75.33± 2.65
G4	76.67± 2.67	75.33± 2.56	76.00± 2.63	75.00± 2.09	76.00± 2.42	75.50± 2.52
G5	76.00± 1.89	74.67± 2.45	75.33± 2.12	75.33± 2.10	75.33± 1.02	75.33± 1.14
Mean	76.39 ± 2.12 a	75.22 ± 2.08 b	75.81± 2.56	75.83± 2.65 a	75.22 ± 1.86 b	75.53± 2.00
F Cultivar	147.00**			3.27*		
F Treatment	22.33**			0.14 ns		
F Cult×Treat	0.30 ns			1.14 ns		

*Different letters within each harvest indicate significant differences (Duncan’s test, p < 0.05). ns, not significant; *p < 0.05; **p < 0.01. Quedl, Quedlinburger; n, 3.

Dry leaf yield (**Table 7**) followed trends similar to dry herbage yield. At H1, G3 (0.84 t ha^-^¹) and G5 (0.79 t ha^-^¹) produced the highest values, while at H2, the maximum value was observed in G3 for “Melis” (0.63 t ha^-^¹).

**Table 7:** Dry leaf yield (t ha^-^¹) of *M. officinalis* as affected by fertilization treatments at two harvest times (H1 and H2) for the cultivars ‘Melis’ and ‘Quedlinburger’.

Treatment	H1 Melis (t ha^-^¹)	H1 Quedl. (t ha^-^¹)	H1 Mean	H2 Melis (t ha^-^¹)	H2 Quedl. (t ha^-^¹)	H2 Mean
G0	0.55± 0.09 e	0.52± 0.07 e	0.54 ± 0.05 e	0.40 de	0.34 ± 0.10 fg	0.37 ± 0.09 d
G1	0.57± 0.06 e	0.57± 0.09 e	0.57 ± 0.09 e	0.37 ef	0.56 ± 0.15 b	0.46 ± 0.11 b
G2	0.73± 0.11 e	0.73± 0.07 e	0.73 ± 0.06 c	0.33 g	0.17 ± 0.07 h	0.25 ± 0.05 f
G3	0.84± 0.12 e	0.84± 0.11 e	0.84 ± 0.08 a	0.63 a	0.43 ± 0.11 cd	0.53 ± 0.12 a
G4	0.66± 0.09 e	0.66± 0.06 e	0.66 ± 0.09 d	0.45 c	0.18 ± 0.04 h	0.31 ± 0.03 e
G5	0.79± 0.11 e	0.78± 0.14 e	0.79 ± 0.11 b	0.42 cd	0.37 ± 0.13 ef	0.39 ± 0.11 c
Mean	0.69 ± 0.10	0.68± 0.12	0.69± 0.09	0.43± 0.11	0.34± 0.07	0.38± 0.12
F Cultivar	2.48 ns			110.05**		
F Treatment	204.87**			382.10**		
F Cult×Treat	0.57 ns			244.35**		

*Different letters within each harvest indicate significant differences (Duncan’s test, p < 0.05). ns, not significant; **p < 0.01. Quedl, Quedlinburger; n, 3.

### 3.4 EO content and yield

EO content ranged from 0.28 to 0.45% DW at H1 and from 0.14 to 0.26% DW at H2 (**Table 8**). At H1, the highest EO content values were obtained in G2 (Animal manure + AS) and G5 (organomineral), ranging between 0.41 and 0.45%. At H2, G2 maintained the highest EO content (0.25–0.26%), whereas G4 (Animal manure + AS + Humic & fulvic acids) and G5 showed lower values (0.14–0.15%).

**Table 8:** EO content (% DW) of *M. officinalis* as affected by fertilization treatments at two harvest times (H1 and H2) for the cultivars ‘Melis’ and ‘Quedlinburger’.

Treatment	H1 Melis (%)	H1 Quedl. (%)	H1 Mean	H2 Melis (%)	H2 Quedl. (%)	H2 Mean
G0	0.32 ± 0.09 e	0.28 ± 0.03 f	0.3 ± 0.08 d	0.14 ± 0.07 d	0.19 ± 0.02 c	0.16± 0.02 d
G1	0.32 ± 0.08 e	0.3 ± 0.08 ef	0.31 ± 0.09 d	0.22 ± 0.04 b	0.22 ± 0.04 b	0.22 ± 0.04 b
G2	0.45 ± 0.02 a	0.41 ± 0.07 bc	0.43 ± 0.2 a	0.25 ± 0.09 a	0.26 ± 0.04 a	0.25 ± 0.09 a
G3	0.36 ± 0.04 d	0.32 ± 0.04 e	0.34 ± 0.02 c	0.19 ± 0.04 c	0.19 ± 0.04 c	0.19 ± 0.04 c
G4	0.4 ± 0.05 bc	0.39 ± 0.04 c	0.4 ± 3.45 b	0.15 ± 0.03 d	0.14 ± 0.02 d	0.15 ± 0.03 e
G5	0.42 ± 0.07 b	0.41 ± 0.03 bc	0.42 ± 0.07 a	0.15 ± 0.03 d	0.15 ± 0.05 d	0.15 ± 0.05 e
Mean	0.38± 0.06 a	0.35 ± 0.02 b	0.37 ± 0.05 d	0.18± 0.06 d	0.19± 0.04	0.19 ± 0.05 d
F Cultivar	96.43**			0.72 ns		
F Treatment	204.02**			193.27**		
F Cult×Treat	4.11*			8.25*		

*Different letters within each harvest indicate significant differences (Duncan’s test, p < 0.05). ns, not significant; *p < 0.05; **p < 0.01. Quedl, Quedlinburger; n, 3.

Cultivar effect was significant at H1, with “Melis” exhibiting higher EO content than “Quedlinburger” (0.38 vs. 0.35%; F = 96.43, p < 0.01). No significant cultivar effect was observed at H2 (F = 0.72, p > 0.05). EO content decreased substantially from H1 to H2 across all treatments.

EO yield (L ha^-^¹), which integrates EO content and dry herbage yield, showed a different ranking of treatments compared to EO content alone (**Table 9**). At H1, G5 produced the highest EO yield (118.1 L ha^-^¹ in “Melis”; treatment mean 116.6 L ha^-^¹), followed by G2 (114.4 L ha^-^¹) and G3 (107.2 L ha^-^¹), while G0 had the lowest value (62.6 L ha^-^¹).

**Table 9:** EO yield (L ha^-^¹) of *M. officinalis* as affected by fertilization treatments at two harvest times (H1 and H2) for the cultivars ‘Melis’ and ‘Quedlinburger’.

Treatment	H1 Melis (L ha^-^¹)	H1 Quedl. (L ha^-^¹)	H1 Mean	H2 Melis (L ha^-^¹)	H2 Quedl. (L ha^-^¹)	H2 Mean
G0	63.60 ± 1.04 ef	61.70 ± 1.33 fg	62.60 ± 3.04 d	22.10 ± 2.33 d	28.10 ± 3.14 bc	25.10 ± 1.56 c
G1	69.00 ± 1.57 e	56.40 ± 0.92 g	62.70 ± 2.06 d	26.90 ± 2.11 c	30.50 ± 3.58 b	28.70 ± 2.17 b
G2	114.40 ± 2.24 ab	114.80 ± 2.11 ab	114.40 ± 3.03 a	30.40± 3.44 b	13.10 ± 1.04 f	21.80 ± 1.99 d
G3	106.90 ± 1.87 c	107.50 ± 1.87 bc	107.20 ± 1.88 b	41.10± 4.01 a	27.20 ± 2.11 c	34.20 ± 3.01 a
G4	95.00 ± 1.24 d	95.60 ± 1.67 d	95.30 ± 2.55 c	29.70± 2.39 bc	7.40± 0.28 g	18.60 ± 1.38 e
G5	118.10 ± 2.98 a	115.00 ± 2.54 a	116.60 ± 1.77 a	30.60± 1.77 b	18.90 ± 1.03 e	24.70 ± 2.12 c
Mean	94.50 ± 2.04 a	91.80 ± 1.98 b	93.10± 2.67	30.10 ± 2.17 a	20.90 ± 2.33 b	25.50± 2.42
F Cultivar	32.91**			1331.57**		
F Treatment	673.92**			179.69**		
F Cult×Treat	7.03*			199.73**		

*Different letters within each harvest indicate significant differences (Duncan’s test, p < 0.05). ns, not significant; *p < 0.05; **p < 0.01. Quedl, Quedlinburger; n, 3.

At H2, the highest EO yield was obtained in G3 (41.1 L ha^-^¹ in “Melis”; treatment mean 34.2 L ha^-^¹). The lowest values were observed in G4 (“Quedlinburger”: 7.4 L ha^-^¹) and G2-Quedlinburger (13.1 L ha^-^¹). The cultivar × treatment interaction was highly significant at H2 (F = 199.73, p < 0.01).

### 3.5 RA content

RA content was significantly affected by fertilization treatments at both harvests (**Table 10**). At H1, the highest RA content was obtained in G5 (63.80 mg g^-^¹ DW in “Melis”; treatment mean 62.47 mg g^-^¹), followed by G3 (54.97 mg g^-^¹) and G4 (51.91 mg g^-^¹). The control treatment (G0) showed the lowest value (36.95 mg g^-^¹). The treatment effect was highly significant (F = 1173.77, p < 0.01), as was the cultivar effect (F = 101.90, p < 0.01), with “Melis” exhibiting higher RA content than “Quedlinburger”.

**Table 10:** RA content (mg g^-^¹ DW) of *M. officinalis* leaf tissue as affected by fertilization treatments at two harvest times (H1 and H2) for the cultivars ‘Melis’ and ‘Quedlinburger’.

Treatment	H1 Melis (mg g^-^¹)	H1 Quedl. (mg g^-^¹)	H1 Mean	H2 Melis (mg g^-^¹)	H2 Quedl. (mg g^-^¹)	H2 Mean
G0	37.43 ± 1.74 h	36.47 ± 1.77 h	36.95 ± 1.14 f	27.13 ± 1.85	26.67 ± 1.68	26.9 ± 11.5 f
G1	40.37 ± 0.06 g	39.97 ± 0.96 g	40.17 ± 1.11 e	30.37 ± 0.06	29.93 ± 0.5	30.15 ± 1.50 e
G2	45.47 ± 0.72 f	44.13 ± 0.35 f	44.80 ± 1.53 d	35.47 ± 0.72	35.1 ± 0.62	35.28 ± 1.38 d
G3	56.5 ± 1.47 c	53.44 ± 2.21 d	54.97 ± 2.01 b	46.5 ± 1.4	46.5 ± 1.4	46.5 ± 2.41 b
G4	53.31 ± 2.45 d	50.52 ± 2.41 e	51.91 ± 1.74 c	41.63 ± 2.45	40.67 ± 2.23	41.15 ± 2.06 c
G5	63.8 ± 1.1 a	61.13 ± 1.15 b	62.47 ± 1.97 a	53.8 ± 1.1	53.03 ± 1.17	53.41 ± 2.80 a
Mean	49.48 ± 1.88 a	47.61 ± 1.64 b	48.54 ± 2.01	39.15 ± 1.67 a	38.65 ± 1.33 b	38.90± 1.84
F Cultivar	101.90**			2.44 ns		
F Treatment	1173.77**			589.92**		
F Cult×Treat	3.88*			0.16 ns		

*Different letters within each harvest indicate significant differences (Duncan’s test, p < 0.05). ns, not significant; *p < 0.05; **p < 0.01. Quedl, Quedlinburger; n, 3.

At H2, RA content remained highest in G5 (53.41 mg g^-^¹ treatment mean), followed by G3 (46.50 mg g^-^¹), whereas G0 showed the lowest value (26.90 mg g^-^¹). The cultivar effect was not significant at H2 (F = 2.44, p > 0.05). Across all treatments, RA content decreased from H1 to H2, although it remained consistently highest under G5 treatment.

### 3.6 Relationship between EO yield and RA content

**Figure 3:**
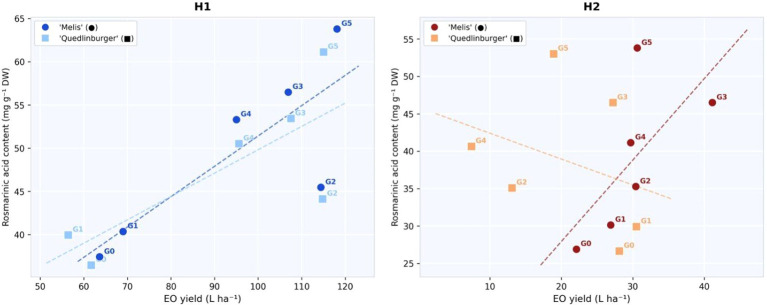
Relationship between EO yield (L ha^-^¹) and RA content (mg g^-^¹ DW) of *Melissa officinalis* L. under six fertilizer treatments at H1 left and H2 right. Circles, ‘Melis’; squares, ‘Quedlinburger’. Dashed lines, linear trend per cultivar. Labels indicate treatment codes (G0–G5).

**Figure 4:**
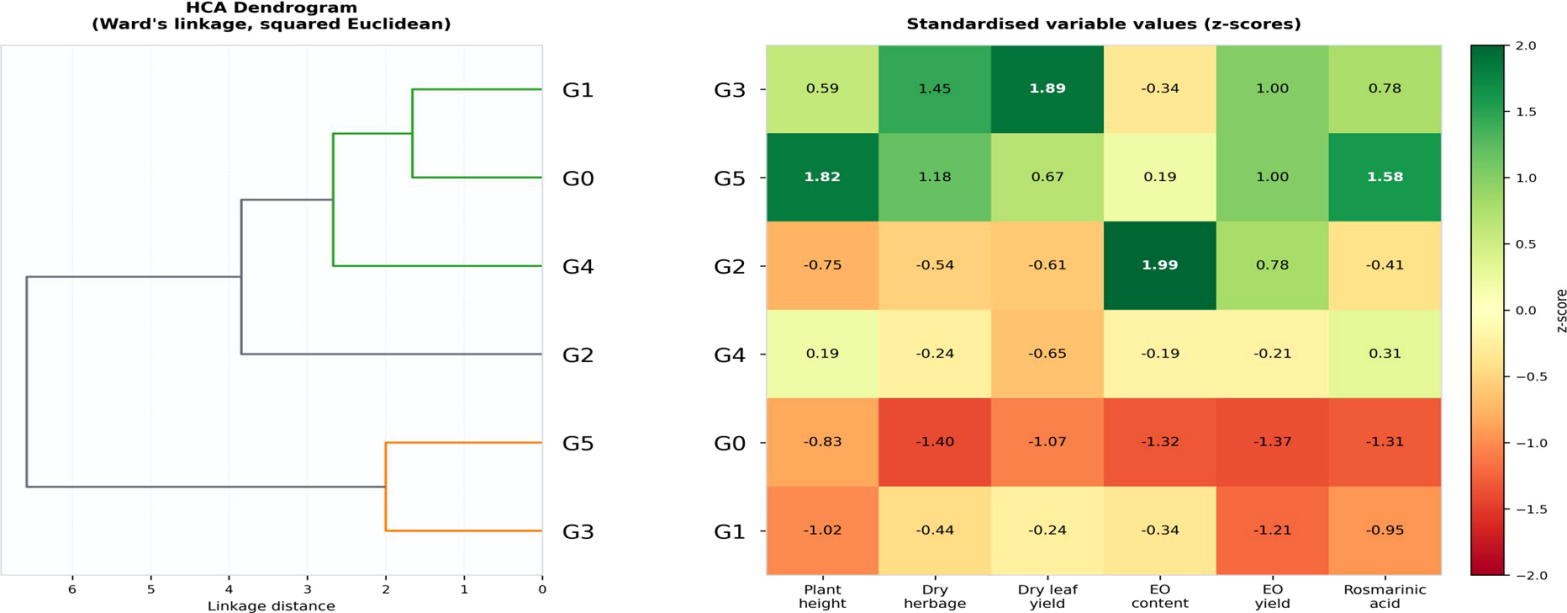
HCA dendrogram (Ward’s linkage; left) and standardized heatmap of *Melissa officinalis* L. fertilizer treatments (right) based on six agronomic and quality parameters (overall treatment means across cultivars and harvests). Heatmap values, z-scores; green, above mean, red, below mean.

The relationship between EO yield and RA content for each treatment × cultivar combination at both harvests is presented in **Figure 3**. At H1, treatments G3 and G5 were positioned in the upper range of both EO yield and RA content, with EO yield values of 107–118 L ha^-^¹ and RA content between 53 and 64 mg g^-^¹. In contrast, the control treatment (G0) was associated with lower EO yield (62–64 L ha^-^¹) and RA content (36–37 mg g^-^¹). At H2, the distribution of treatments differed from that observed at H1. G3 maintained relatively high RA content, while EO yield values were lower compared to H1. Among treatment × cultivar combinations, the lowest values were observed in G2-Quedlinburger (13.1 L ha^-^¹; 35.10 mg g^-^¹). Trend lines shown in **Figure 4** illustrate differences in the EO-RA relationship between harvests and among treatments.

### 3.7 Multivariate Analysis (PCA and HCA)

**Figure 5:**
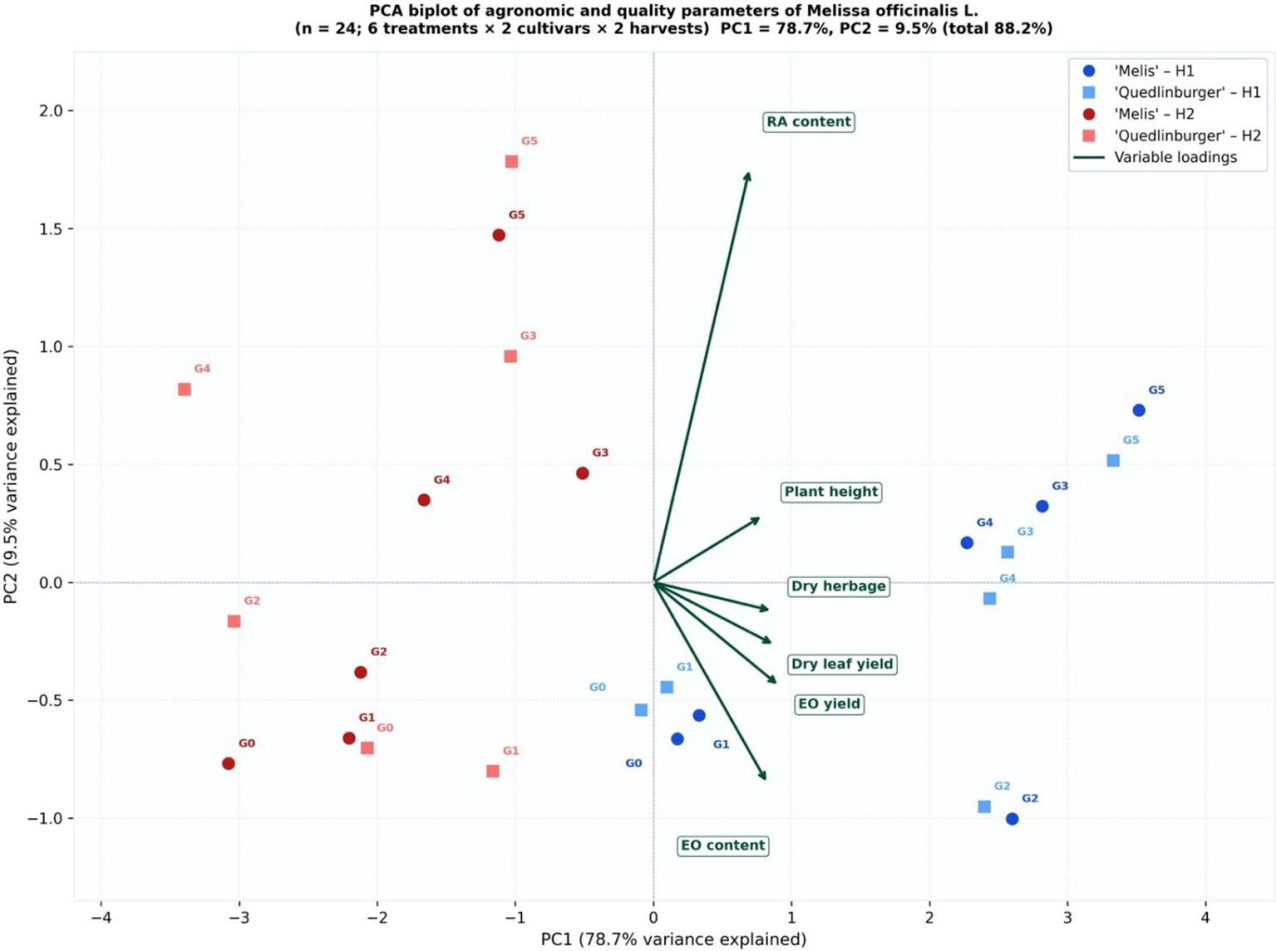
PCA biplot of agronomic and quality parameters of *Melissa officinalis* L. under six fertilizer treatments in two cultivars at two harvest times. n, 24 observations, PC1, 78.7%, PC2 = 9.5% (total 88.2% variance explained). Circles, ‘Melis’; squares, ‘Quedlinburger’. Dark blue/dark red, H1; light blue/light red, H2. Variable loadings shown as green arrows.

Principal component analysis (PCA) of the standardized 24 × 6 dataset (6 treatments × 2 cultivars × 2 harvests; 6 variables) explained 88.2% of the total variance, with PC1 accounting for 78.7% and PC2 for 9.5% (**Figure 5**). PC1 showed positive loadings for RA content, EO yield, dry herbage yield, and dry leaf yield, and negative loadings for EO content. PC2 was mainly associated with plant height and differences between harvest times. Treatments G5 and G3 exhibited the highest positive scores along PC1 in both cultivars, whereas G0 was located at the lower end of the axis. Observations from H1 and H2 were separated along PC2.

Hierarchical cluster analysis (HCA) based on treatment mean values (Ward’s linkage) identified three major clusters (**Figure 4**). Cluster I consisted of G0, characterized by the lowest values across the evaluated parameters. Cluster II included G1, which showed moderate yield levels and relatively lower RA content among fertilized treatments. Cluster III comprised G2, G3, G4, and G5, associated with higher yield and quality parameters.

Within Cluster III, G5 and G3 formed a closely related sub-cluster, corresponding to the highest values of RA content and EO yield. The heatmap (**Figure 4**) further illustrates that G5 and G3 exhibited the highest positive standardized (z-score) values for RA content and EO yield, whereas G0 showed the lowest values.

## 4 Discussion

The results of this study provide key insights into the agronomic management of *M. officinalis* under Mediterranean conditions. First, fertilization strategies incorporating organic matter and humic substances consistently performed better than mineral nitrogen alone across multiple yield and quality parameters. Second, EO content and RA exhibited contrasting trends from H1 to H2, although organic-enriched treatments promoted relatively high levels of both parameters at H1. Third, humic acid supplementation appears to play an important role in sustaining second-harvest yield under warm and drought-prone conditions. Finally, cultivar responses were strongly dependent on fertilization treatments, particularly at the second harvest stage.

### 4.1 Herbage yield: role of humic substances under drought conditions

G3 (ammonium sulfate combined with humic and fulvic acids) produced the highest fresh herbage yield at H1 (15.41 t ha^-^¹ in “Quedlinburger”), highlighting the importance of organic-enriched fertilization under the prevailing soil conditions. The experimental soil, characterized by sandy texture (77-79% sand), low organic matter (0.80-1.04%), low available K, and slightly alkaline pH (7.14-7.39), represents a system with limited nutrient retention and water-holding capacity.

Under such conditions, humic and fulvic substances are known to enhance nutrient availability through cation complexation and to improve root development and rhizosphere activity (Nardi et al., 2002; Canellas et al., 2015). These effects may be particularly relevant in alkaline soils, where micronutrients are prone to precipitation and reduced availability (Barzgar et al., 2025). In addition, humic substances have been reported to stimulate root growth and increase the effective soil volume explored by plants, potentially improving both nutrient and water uptake (Canellas and Olivares, 2014; Andrade et al., 2023).

The relevance of these mechanisms becomes more apparent under the drought conditions observed during the 2024 growing season, when precipitation deficits coincided with the regrowth period between harvests. In this context, the markedly lower H2 yield observed in G2- Quedlinburger (2.37 t ha^-^¹), compared to the corresponding control, suggests that manure alone may have been insufficient to sustain regrowth under water-limited conditions. In contrast, treatments including humic substances (G3 and G4) maintained relatively higher productivity. These findings may indicate that humic substances may contribute to improved plant performance under combined nutrient and water limitations, particularly in coarse-textured, low-organic-matter soils. However, the absence of post-harvest soil analyses limits direct verification of nutrient mobilization processes, and future studies should incorporate soil chemical and biological indicators to further elucidate these mechanisms.

### 4.2 EO yield: organomineral fertilization and harvest timing

The high EO yield obtained under G5 at H1 (118.1 L ha^-^¹ in “Melis”) is consistent with values reported for well-managed field-grown *M. officinalis* (Mafakheri et al., 2016). The organomineral 12-12–12 formulation provides a balanced nutrient supply that may support monoterpene biosynthesis via the MEP pathway, while avoiding excessive nitrogen inputs that could shift carbon allocation toward primary metabolism (Gershenzon, 1994).

The substantial decline in EO content from H1 to H2 aligns with previously reported seasonal patterns under Mediterranean conditions (Moradkhani et al., 2010). In the present study, this decline may have been intensified by the elevated temperatures observed during June-July 2024, which likely accelerated glandular trichome senescence and EO volatilization (Sangwan et al., 2001). These results highlight the importance of harvest timing for EO-oriented production systems. In particular, the first harvest (H1) appears to be critical for maximizing EO yield under Mediterranean climatic conditions. Similar responses to fertilization and harvest timing have been reported in other Lamiaceae species, including peppermint (Fallah et al., 2024b).

### 4.3 RA: nutrient-mediated regulation and interaction with EO yield

The substantial increase in RA content under G5 (69-99% relative to G0) across both harvests highlights the strong responsiveness of phenylpropanoid metabolism to fertilization strategy. RA is synthesized via the phenylpropanoid pathway, with phenylalanine ammonia-lyase (PAL) representing the key rate-limiting step (Petersen et al., 2010). PAL activity is known to be influenced by nitrogen availability, micronutrient supply, and environmental conditions, linking nutrient status to secondary metabolite accumulation (Sofo et al., 2016; Hassan et al., 2020).

These findings can be interpreted within the framework of the carbon–nutrient balance (CNB) hypothesis, which predicts enhanced phenolic accumulation under conditions of adequate but non-excessive nitrogen availability (Bryant et al., 1983; Gershenzon, 1994). In this study, the organomineral treatment (G5), combining balanced NPK supply with organic matter, may have maintained a nutrient regime that supported both primary growth and phenylpropanoid flux. The consistently higher RA levels observed in G5 compared to G1 (mineral nitrogen alone) support the role of nutrient form and release dynamics in regulating secondary metabolism. Mineral nitrogen applied alone may preferentially promote primary growth and biomass accumulation, whereas organomineral fertilization provides a more gradual nutrient supply that can favor carbon partitioning toward the phenylpropanoid pathway and rosmarinic acid biosynthesis. Similar trends have been reported in *Melissa officinalis* and other RA-producing medicinal plants, where organic nutrient sources significantly enhanced secondary metabolite accumulation compared with mineral-only fertilization (Fallah et al., 2024b). In support of this interpretation, previous studies have also shown that excessive or rapidly available mineral nitrogen may suppress phenylpropanoid metabolism and reduce RA accumulation. For example, in Ocimum basilicum, high mineral nitrogen doses significantly down-regulated RA levels (Kolega et al., 2020).

The decrease in RA content from H1 to H2 across all treatments further suggests a pronounced phenological and harvest-time effect. At later growth stages, plants may increasingly allocate carbon resources toward reproductive development and storage processes rather than toward metabolically expensive secondary metabolites such as RA (Gomes et al., 2023). In addition, late-season environmental stressors, particularly elevated temperature and radiation intensity, may contribute to oxidative degradation of existing phenolic pools (Fallah et al., 2024b). This interpretation is further supported by previous findings in *Salvia officinalis*, where the first harvest consistently yielded the highest RA concentrations, while subsequent harvests failed to maintain these levels (Habán et al., 2019).

The relationship between EO yield and RA content provides additional insight. At H1, treatments G3 and G5 were associated with high values of both EO yield and RA content, suggesting that organic-enriched fertilization can support simultaneous enhancement of terpenoid and phenylpropanoid pathways. In contrast, the divergence observed from H1 to H2, characterized by declining EO and increasing RA, likely reflects a possible phenological shift in carbon allocation between these pathways.

### 4.4 Multivariate quality framework

The strong contribution of PC1 (78.7%), driven by RA content, EO yield, and dry leaf yield, together with the positive positioning of G5 and G3 along this axis, indicates that these treatments were associated with both high productivity and quality-related traits. This finding suggests that, under the conditions of this study, organic-enriched fertilization can support simultaneous improvement of yield and pharmacologically relevant quality parameters. Organic fertilization has been widely reported to enhance both yield and essential oil quality in aromatic plants. In lemon balm (*Melissa officinalis*), application of poultry, sheep, and cattle manure increased biomass by 41–60% and essential oil yield by 60–71%, while also improving the proportions of key constituents such as neral and geranial and enhancing antioxidant capacity (Fallah et al., 2024). Similar trends have been observed in oregano (*Origanum onites*), where farmyard manure and spent mushroom compost increased fresh yield by up to 13.59% and essential oil content by 18.8–50.1% compared to untreated controls (Asri, 2023). In Greek sage (*Salvia fruticosa*), organic fertilization supported essential oil yields as high as 316.89 L ha^-^¹, demonstrating an effective balance between biomass production and secondary metabolite accumulation (Asri et al., 2023). Likewise, in lemongrass (*Cymbopogon flexuosus*), quail manure application elevated both plant weight and total essential oil yield, with a specific increase in neral content (Lopes et al., 2019).

The grouping of G5 and G3 within the same cluster in HCA further supports their functional similarity, despite differences in nitrogen source. This pattern points to the potential role of organic matter and humic substances in enhancing overall plant performance beyond mineral nitrogen supply.

### 4.5 Cultivar × fertilization interaction

The significant cultivar × fertilization interactions observed across multiple parameters demonstrate that treatment responses were strongly genotype-dependent, particularly under the environmental constraints of the second harvest (H2). While interaction effects were weak or absent during the first harvest (H1), they became consistently significant in H2 across plant height, fresh and dry herbage yield, dry leaf yield, and essential oil yield, indicating that stress conditions amplified genotype-specific responses. This aligns with findings in other medicinal plants where environmental conditions significantly modulate genotypic expression, impacting not only biomass but also the accumulation of bioactive compounds (Németh et al., 2019). For instance, water deficiency has been shown to differentially affect essential oil content in various *Melissa officinalis* genotypes, highlighting the intricate interplay between environmental stressors and cultivar-specific metabolic pathways (Szabó et al., 2017).

“Melis” exhibited a more stable and resilient performance across treatments during H2, whereas “Quedlinburger” showed greater variability and sensitivity to fertilization regimes. This contrast was particularly evident under G2, where fresh herbage yield declined to 2.37 t ha^-^¹ in “Quedlinburger” compared to 6.48 t ha^-^¹ in “Melis”, clearly illustrating a genotype-specific response under combined nutrient and water stress. A similar pattern was observed for essential oil yield, which decreased to 13.10 L ha^-^¹ in “Quedlinburger” under G2, while remaining substantially higher in “Melis” (30.40 L ha^-^¹). These differential responses underscore the necessity of genotype-specific management practices, as optimal fertilizer recommendations can vary significantly between cultivars depending on environmental conditions (Oliveira et al., 2021; Yousefi et al., 2025).

In contrast, treatments incorporating integrated nutrient inputs, particularly G3 and G5, resulted in more balanced and consistent responses between cultivars. For instance, under G3 in H2, fresh herbage yield reached 8.14 t ha^-^¹ in “Melis” and 6.48 t ha^-^¹ in “Quedlinburger”, indicating a reduced performance gap compared to stress-prone treatments. These patterns suggest a potential stabilizing effect of combined nutrient sources under drought-prone conditions. Moreover, the biosynthesis and accumulation of essential oils, terpenes, and phenolic compounds in plants are frequently amplified under stress, suggesting their role as stress mediators and indicators of genotype-specific physiological resilience (Gomes et al., 2023; Mugwanya et al., 2025).

Interestingly, leaf ratio was not affected by cultivar × fertilization interaction, remaining within a narrow range (~75–77%) across treatments, indicating that structural biomass allocation between leaves and stems was relatively stable. This suggests that the observed differences in yield were primarily driven by physiological or metabolic responses rather than morphological changes. This emphasizes that while external morphology may remain consistent, the internal physiological and biochemical pathways, such as altered photosynthetic activity or recalibrated secondary metabolism, are key determinants of yield variation under varied environmental and fertilization regimes (Alipour et al., 2025). Essential oil yield showed strong interaction patterns, whereas rosmarinic acid (RA) content was mainly treatment-driven, with limited interaction effects in H2, highlighting differences in how primary and secondary metabolism respond to genotype and fertilization. This divergence suggests that the physiological mechanisms governing essential oil production are more susceptible to genotype-environment interactions than those regulating rosmarinic acid biosynthesis, potentially due to distinct enzymatic pathways or compartmentalization strategies within the plant (Ciriello et al., 2021).

Overall, these findings emphasize that fertilization strategies cannot be evaluated independently of genotype, as cultivar-specific responses play a key role in determining both yield and quality outcomes under Mediterranean conditions.

### 4.6 Cultivar selection and climate resilience

The two cultivars exhibited distinct performance patterns across harvests. “Melis” showed higher EO content and RA levels at H1 and maintained comparatively higher productivity at H2, indicating a more stable response under the prevailing conditions.

The relatively low available potassium (K) levels in the experimental soil (66–75 mg kg^-^¹; **Table 2**) may have influenced cultivar-specific responses, as potassium plays a key role in stomatal regulation and plant water relations. Under such conditions, differences in cultivar sensitivity to water stress may become more pronounced. K serves as the primary osmoticum in plant cells, regulating turgor pressure, stomatal movement, and water use efficiency (Ashraf et al., 2002; Jordan-Meille et al., 2018; Mir et al., 2019). Its role becomes particularly critical under drought and heat stress, as K facilitates ion flux in guard cells, directly governing photosynthetic capacity and stomatal conductance (Mir et al., 2019; Akhtar et al., 2023). K deficiency impairs stomatal closure under high evaporative demand, leading to excessive water loss, while reduced soil moisture further limits K^+^ diffusion to roots, compounding drought-related productivity losses (Shahzad et al., 2017; Jordan-Meille et al., 2018). K-efficient genotypes maintain superior turgor and gas exchange under water-limited conditions compared to sensitive counterparts (Wei et al., 2013; Pacheco et al., 2021), and adequate K supply has been shown to stabilize mineral content and support biomass accumulation under stress in both medicinal plants and cereals (Ashraf et al., 2002; Kostopoulou et al., 2015).

The climatic conditions of 2024, characterized by elevated temperatures and reduced precipitation during the growing season, provide a relevant context for interpreting these results. In this environment, fertilization strategies that combine mineral nutrients with organic inputs (e.g., G3 and G5) were associated with more stable performance across harvests. These findings may have implications for the selection of fertilization practices and cultivars under increasingly variable Mediterranean climate conditions.

### 4.7 Limitations of the study

This study has several limitations that should be considered when interpreting the results. First, direct measurements of soil moisture dynamics and plant water potential were not performed, which limited our ability to precisely quantify plant water status, particularly under the elevated temperature and reduced precipitation conditions observed during the second harvest period. Second, although essential oil yield was determined, detailed compositional analysis of EO constituents was not included in the present evaluation. In addition, leaf mineral nutrient concentrations and photosynthetic parameters (e.g., chlorophyll content, stomatal conductance, and photosynthetic rate) were not measured, restricting the physiological interpretation of treatment effects. Furthermore, enzymatic activities and gene expression analyses related to phenylpropanoid and terpenoid biosynthesis pathways were beyond the scope of this study. Future research integrating these physiological and molecular parameters would provide a more comprehensive understanding of the mechanisms underlying yield and quality responses in *Melissa officinalis*.

## 5 Conclusions

This study indicates that fertilization strategy may play an important role in shaping both agronomic performance and phytochemical quality in *Melissa officinalis* under Mediterranean field conditions. In particular, integrated nutrient management approaches combining mineral and organic inputs appeared to be more effective than mineral fertilization alone in supporting biomass production and the accumulation of pharmacologically relevant metabolites. The relatively better performance observed under organomineral and humic-enriched treatments suggests that nutrient form and release dynamics may contribute to the regulation of essential oil productivity and rosmarinic acid accumulation, especially under increasingly warm and water-limited environments. The more stable second-harvest response of the ‘Melis’ cultivar further suggests its potential suitability for dual-harvest production systems in Mediterranean climates. These findings may provide a useful basis for developing more sustainable nutrient management strategies for medicinal and aromatic plant production under Mediterranean and heat and water deficit agroecosystems. Future studies should integrate detailed essential oil compositional profiling, physiological measurements, nutrient dynamics, and molecular analyses to better elucidate the mechanisms underlying fertilizer-driven changes in yield and secondary metabolism.

## Data Availability

The raw data supporting the conclusions of this article will be made available by the authors, without undue reservation.
